# Chemical Characterization and Assessment of the Neuroprotective Potential of *Euphrasia officinalis*

**DOI:** 10.3390/ijms252312902

**Published:** 2024-11-30

**Authors:** Antonis Ververis, Sotiris Kyriakou, Hariklia Paraskeva, Mihalis I. Panayiotidis, Michael Plioukas, Kyproula Christodoulou

**Affiliations:** 1 Neurogenetics Department, The Cyprus Institute of Neurology and Genetics, Nicosia 2371, Cyprus; antonisv@cing.ac.cy (A.V.); charicleaparaskeva@gmail.com (H.P.); 2 Department of Cancer Genetics, Therapeutics and Ultrastructural Pathology, The Cyprus Institute of Neurology and Genetics, Nicosia 2371, Cyprus; sotirisk@cing.ac.cy (S.K.); mihalisp@cing.ac.cy (M.I.P.); 3 Department of Life and Health Sciences, School of Sciences and Engineering, University of Nicosia, Nicosia 2417, Cyprus; pmichael.gr@hotmail.com

**Keywords:** *Euphrasia*, eyebright, extracts, amyloid, neuroprotection

## Abstract

*Euphrasia officinalis* L., commonly known as eyebright, is a medicinal plant used in folk medicine for eye disorders and memory loss. Due to its abundance of compounds with proven neuroprotective properties, there has been growing interest in exploring eyebright’s potential health benefits, particularly for preventing or treating neurodegenerative diseases like Alzheimer’s disease. Here, seven distinct extracts were generated using solvents of different polarities, consecutively, from plants grown in Greece. The extracts were chemically characterized and assessed for their antioxidant, anticholinesterase, and anti-neurotoxic potentials. Our findings demonstrated eyebright’s notable antioxidant capacity with five extracts exhibiting significant anti-neurotoxic properties by enhancing cell viability by 17.5 to 22.6% in human neuroblastoma cells exposed to neurotoxic amyloid-beta peptides. The ethyl acetate and butanolic extracts were the most effective across all assays, likely due to their high concentrations of active compounds. Therefore, eyebright may be harnessed for developing functional foods, supplements, and pharmaceuticals with potential benefits against Alzheimer’s disease. This study marks the first identification of neuroprotective properties in a *Euphrasia* species, highlighting its broader therapeutic potential and paving the way for further research.

## 1. Introduction

In 1906, German neuropathologist Alois Alzheimer first described Alzheimer’s disease (AD) as a debilitating age-related neurodegenerative condition with an unknown cause [1]. AD is the most prevalent form of dementia, characterized by cognitive impairments, notably memory loss and confusion, which progressively worsen over time. As Alzheimer’s disease progresses, patients face increasing difficulties with communication, eating, and sleep, which ultimately lead to a decline in overall health and, eventually, death [2]. Consequently, AD profoundly impacts patients, their families, and society, particularly by impairing instrumental activities of daily living, such as financial management. Studies in the Greek geriatric population reveal that even mild AD can lead to deficits in judgment, problem-solving, and complex decision-making, all crucial for managing personal finances [3]. Families, often acting as informal caregivers, face increased responsibilities as well as emotional and financial burdens, while society bears the healthcare costs and the need to protect patients from financial exploitation [4,5].

The primary neuropathological features of AD include extracellular amyloid plaques and intracellular neurofibrillary tangles, accompanied by neuronal loss, oxidative stress, and neuroinflammation [6]. Until recently, treatment options were limited to cholinesterase inhibitors and N-methyl-D-aspartate receptor antagonists, providing symptomatic care, primarily by partly improving cognitive function, but not altering the disease’s onset or progression. In 2021, the Food and Drugs Administration (FDA) granted accelerated approval to aducanumab, a novel drug designed to slow AD progression. Aducanumab is a monoclonal antibody that targets amyloid beta (Aβ) fibrils. If the amyloid cascade hypothesis is true, which suggests that amyloid-beta accumulation is the primary pathogenic cause of AD, this drug could potentially slow or halt disease onset and progression by reducing amyloid-beta aggregates [7]. However, aducanumab’s high cost and concerns over its efficacy and safety limited its success [8,9]. More recently, the FDA granted full approval to lecanemab, another monoclonal antibody shown to reduce amyloid burden and slow cognitive decline in patients with early AD. Nevertheless, its efficacy and safety remain to be clarified in patients at other stages of the disease [10]. Therefore, ongoing research is essential to discover affordable, effective, and safe therapeutic alternatives.

Oxidative stress plays a critical role in the pathophysiology of AD. It is associated with aging and occurs when a biological system cannot manage elevated reactive oxygen species (ROS) levels. In AD, oxidative stress can trigger and intensify amyloid beta aggregation and neurofibrillary tangle build-up, which, in turn, intensify oxidative stress and accelerate neuronal death, creating a vicious cycle [11,12]. Moreover, the brains of AD patients exhibit reduced levels of acetylcholine, a neurotransmitter that plays a crucial role in ROS detoxification under oxidative stress and provides neuroprotection against Aβ toxicity [13,14]. Cholinesterase enzymes, including acetylcholinesterase (AChE) and butyrylcholinesterase (BChE), degrade acetylcholine, and their levels are elevated in the brains of AD patients [15]. In addition, AChE enhances amyloid fibrillation by forming cytotoxic AChE-Aβ complexes [16]. Thus, a promising approach could involve developing medications, functional foods, and supplements with anti-amyloid, antioxidant, and anticholinesterase properties to slow the progression or delay the onset of AD.

Medicinal plants represent a rich source of bioactive compounds and extracts that are currently the focus of extensive research. The aim is to determine whether these substances have the potential to impede or halt the progression of various pathological conditions and reduce the incidence of disease onset. In the case of AD, medicinal plants could offer a cost-effective alternative, while mitigating the possible side effects associated with conventional drugs. Owing to their complex nature, many natural compounds and extracts may act on multiple targets within the brain and throughout the body, potentially providing broader therapeutic effects compared to synthetic drugs, which usually focus on a single biological pathway [6,17]. These herbal products could serve as functional foods, providing nutritional benefits and specific health advantages. They may help prevent or delay the onset and progression of various pathological conditions, including AD, thereby supporting overall wellness [18].

Eyebright, scientifically known as *Euphrasia officinalis* L. (EO), is an annual, non-woody, hemiparasitic plant that has historically been used in herbal medicine to treat various eye conditions, such as conjunctivitis [19]. Belonging to the Orobanchaceae family, EO is found across Europe, Asia, and the United States, and serves as a source of bioactive compounds, including phenolics, flavonoids, and iridoids [20]. These compounds contribute to EO’s significant biological properties, such as antioxidant, anti-inflammatory, antimicrobial, anti-cancer, antihyperglycemic, anti-catarrhal, and antiaging effects, which can be beneficial in treating a wide range of disorders [19,20,21,22,23]. Traditionally, eyebright has been used for the treatment and prevention of eye disorders [23], and in folk medicine to address memory-related issues [24,25], a hallmark symptom of AD [26]. However, despite its content of tannins, iridoids, and flavonoids, compounds known to enhance memory and cognition [27], no published studies have confirmed its neuroprotective effects. Given EO’s established antioxidant and anti-inflammatory properties, which are particularly valuable for treating neurodegenerative conditions like AD, we aimed to investigate whether EO also possesses anti-neurotoxic potential [28].

This study aims to experimentally confirm, for the first time, the potential neuroprotective properties of EO, a plant used in folk medicine to treat memory impairments. To achieve this, we generated seven distinct EO extracts using different solvents and conducted chemical characterization to identify the unique composition of each extract. We then evaluated their antioxidant, anticholinesterase, and anti-neurotoxic properties to assess the neuroprotective potential of these extracts. 

## 2. Results

### 2.1. EOEA and EOB Are Rich in Phenolics and Flavonoids

**Figure 1:**
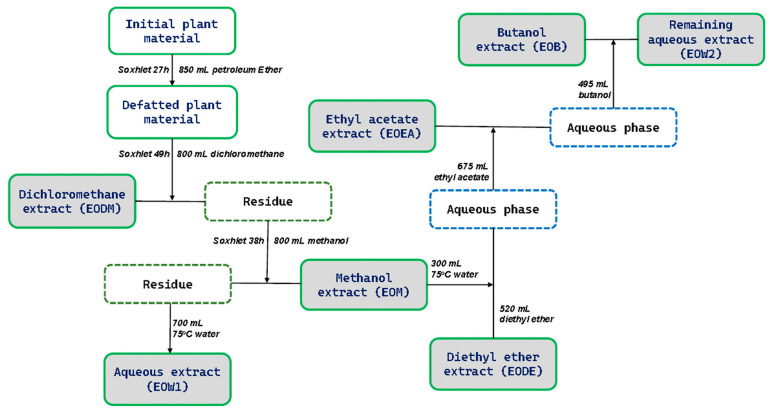
Graphical representation of the extraction process used to produce seven distinct EO extracts.

Initially, we generated the extracts used in this study. After defatting with petroleum ether, the plant material was consecutively extracted with solvents of increasing polarity. All extracts were evaporated to dryness under a vacuum. Extraction began with dichloromethane, yielding the dichloromethane extract (EODM). The next solvent used, methanol, formed the methanolic extract (EOM), and finally, water yielded the aqueous extract (EOW1). EOM was further partitioned sequentially with diethyl ether (EODE extract), ethyl acetate (EOEA extract), and butanol (EOB extract). Finally, the remaining aqueous extract (EOW2) was collected (Figure 1). 

The ethyl acetate and the butanolic extracts showed relatively higher levels of phenolics (405.98 ± 16.54 and 236.89 ± 12.68 mg of gallic acid equivalents per gram, respectively) and flavonoids (766.98 ± 10.13 and 651.33 ± 12.21 μmol catechin equivalents per gram, respectively) than the other extracts. The methanolic extract displayed a significant flavonoid content, followed by the diethyl ether, the initial aqueous, and the remaining aqueous extracts, while the dichloromethane extract contained the lowest flavonoid concentration (22.51 ± 1.01 mg of gallic acid equivalents per gram). Similar trends were observed in total phenolic content, with EOM, EODE, EOW1, and EOW2 trailing EOEA and EOB, while EODM showed the lowest phenolic content (24.65 ± 1.23 μmol catechin equivalents per gram) (Table 1).

### 2.2. EOEA and EOB Are Rich in the Identified Polyphenols

**Figure 2:**
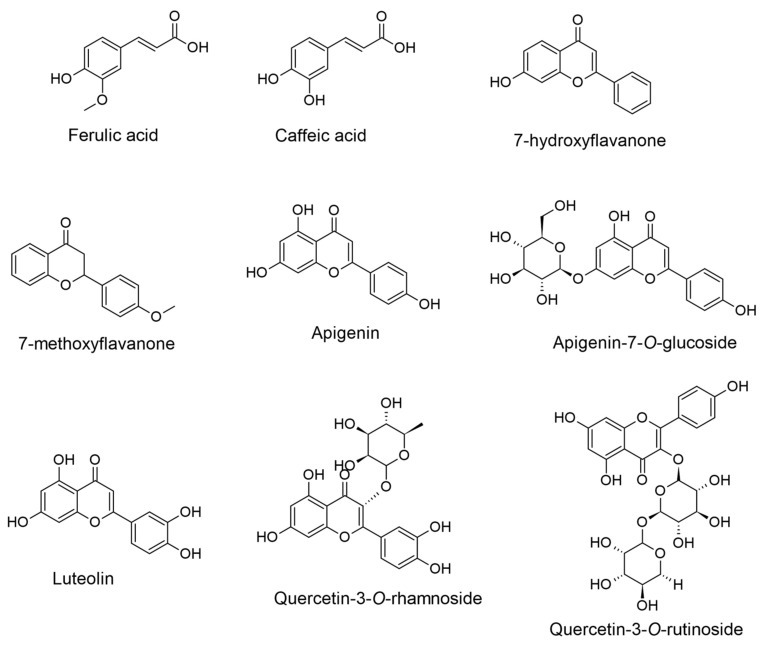
The chemical structures of the major compounds identified in both EOEA and EOB extracts.

A total of forty-three compounds were identified and quantified in the EO extracts using ultra-performance liquid chromatography-tandem mass spectrometry (UPLC-MS/MS). Aligned with the results for total phenolic and flavonoid content, EOEA and EOB together displayed notably high concentrations of key constituents, including ferulic acid, caffeic acid, 7-hydroxyflavanone, 4′-methoxyflavanone, apigenin, apigenin-7-*O*-glucoside, luteolin, quercetin-3-*O*-rhamnoside, and quercetin-3-*O*-rutinoside (Figure 2) (Table 1). Additionally, EOEA was particularly rich in protocatechuic acid, gallic acid, ethyl gallate, dihydrocaffeic acid, chlorogenic acid, and myricetin-3-*O*-galactoside. Finally, apigenin and apigenin-7-*O*-glucoside were especially abundant in EODE (Table 1).

### 2.3. EOEA and EOB Show Remarkable Antioxidant Potential

The EOEA extract showed the most potent free radical scavenging ability among all tested extracts in the DPPH (2,2-diphenyl-1-picrylhydrazyl) assay, showing activity nearly equivalent to the positive control Trolox (antioxidant efficiency (AE) = 5.59). The aqueous, methanolic, diethyl ether, remaining aqueous, and butanolic extracts also demonstrated a noteworthy capacity for scavenging free radicals. In contrast, the dichloromethane extract exhibited negligible ability in scavenging free radicals (Table 2).

Similar to the DPPH assay, EOEA exhibited the strongest reducing activity in the FRAP (ferric reducing activity power) assay, followed by the EOB extract that showed remarkable potential, and the EOM, EODE, and EOW1 extracts. The EOW2 and EODM extracts exhibited the lowest efficiency in the FRAP assay (Table 2).

**Figure 3:**
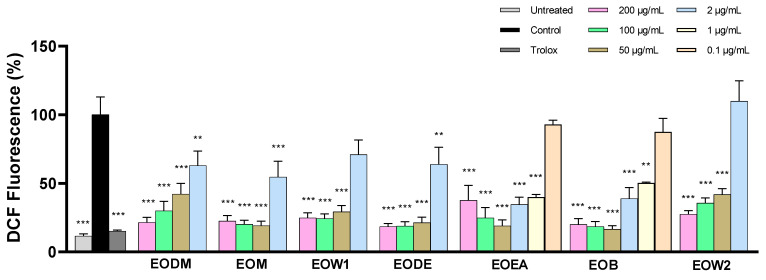
The antioxidant potency of EO fractions was assessed using the DCFDA assay in SH-SY5Y cells incubated with H_2_O_2_. Every group was treated with 50 μΜ H_2_O_2_ except untreated cells. The error bars show the SEM (standard error of the mean) of six independent tests. ** points to *p* < 0.01 and *** to *p* < 0.001 statistical importance, in comparison to control cells.

A similar trend was observed in the DCFDA (dichlorofluorescein diacetate) assay, performed in SH-SY5Y cells treated with hydrogen peroxide. EOEA and EOB were the most effective extracts in reducing ROS, comparable to Trolox, followed by the other extracts (Figure 3, Table 2). A range of extract concentrations was used for the calculation of IC_50_ and most extracts showed strong antioxidant ability at concentrations between 50–200 μg/mL, as determined by a one-way analysis of variance test followed by Dunnett’s multiple comparison test (Table S1). The antioxidant effect began to diminish at concentrations of 2 μg/mL and lower across all extracts.

Conclusively, the EOEA extract was the most potent antioxidant among the EO extracts tested, comparable to Trolox, followed by EOB. The EODM and EOW2 extracts exhibited the weakest antioxidant activity. Given that the three assays assess different aspects of antioxidant ability, it is reasonable to conclude that EOEA and EOB exert their antioxidant effects through various mechanisms, such as quenching free radicals or utilizing ferric-reducing capacity.

### 2.4. EO Extracts Demonstrate Anticholinesterase Activity

The EO extracts exhibited relatively weak anti-acetylcholinesterase activity, with EOB showing the strongest effect. It was the only extract to present an IC_50_ value below 1 mg/mL and an IC_25_ value below 0.5 mg/mL. The other extracts demonstrated weaker activity, with EOW2 being inactive (Table 3). 

For anti-butyrylcholinesterase activity, EOB, EOEA, EODM, and EODE exhibited the most potent effects, while EOW1 and EOW2 showed the least activity. In this case, all extracts presented with an IC_50_ value below 1 mg/mL, except for the two aqueous ones (Table 3).

### 2.5. Five EO Extracts Exhibit Important Anti-Neurotoxic Potential

**Figure 4:**
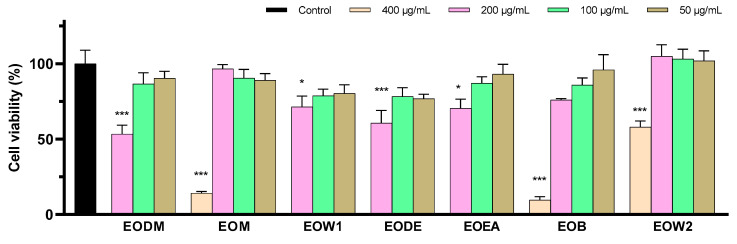
The maximum non-toxic concentration of ΕO extracts on SH-SY5Y cells was estimated using the MTT assay. Error bars depict the SEM of four independent tests. * indicates statistical significance at *p* < 0.05, and *** at *p* < 0.001, when compared to control untreated cells.

Initially, the cytotoxic effect of the EO extracts in SH-SY5Y cells was investigated to detect the maximum non-toxic concentrations of the extracts. These concentrations served as the starting dilutions for which EO extracts would be used for the anti-neurotoxicity assessment. EODM, EOW1, EODE, and EOEA were found to be the most toxic, showing statistically significant cytotoxicity in concentrations ≥200 μg/mL. EOM, EOB, and EOW2 demonstrated a similar effect in concentrations ≥400 μg/mL (Figure 4, Table S2).

**Figure 5:**
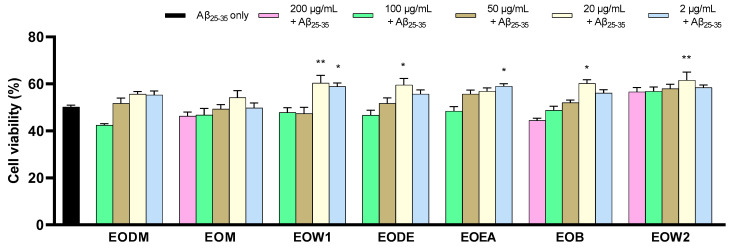
The neuroprotective ability of the EO extracts against Aβ toxicity in SH-SY5Y cells was estimated using the MTT assay. Error bars depict the SEM of five independent tests. * indicates statistical importance at *p* < 0.05, and ** points to *p* < 0.01, when compared with cells treated only with 30 μΜ Aβ_25-35_ (Control).

Subsequently, serial dilutions of the extracts were prepared at concentrations below the cytotoxicity threshold and used to treat SH-SY5Y cells incubated with neurotoxic Aβ_25-35_ peptides. At a concentration of 30 μM, Aβ_25-35_ aggregates reduced cell viability by approximately 50%. When treated with 20 μg/mL and 2 μg/mL of EOW1, 20 μg/mL of EODE, EOB, EOW2, and 2 μg/mL of EOEA, cells showed a mild but statistically significant improvement in viability, with values increasing from 17.5% to 22.6% in Aβ_25-35_-treated cells. None of these five extracts exhibited significant anti-neurotoxic effects at the other concentrations tested. EODM and EOM did not demonstrate any anti-neurotoxic effects at all concentrations evaluated. This suggests that certain extracts may exert a protective effect against Aβ_25-35_-induced neurotoxicity, but the efficacy is limited to specific concentrations, and the activity varies across different extracts (Figure 5, Table S3).

## 3. Discussion

To our knowledge, this is the first work investigating the possible health-beneficial potential of a *Euphrasia* species for Alzheimer’s disease. Here, we studied one of the more common species of the genus, *E. officinalis*, by partitioning plant material from the aerial parts into seven distinct extracts using solvents of different polarities. These extracts were chemically characterized and investigated for their antioxidant, anticholinesterase, and anti-neurotoxic capacities. We have demonstrated for the first time that specific EO extracts possess a neuroprotective potential toward amyloid-beta neurotoxicity in neuroblastoma cells.

The chemical characterization of the EO extracts confirmed the distinct character of all the extracts since each one showed a different chemical profile. Certain compounds were identified that are known as major compounds of *Euphrasia spp.*, such as phenolic acids (gallic acid, caffeic acid, ferulic acid), flavones (apigenin, luteolin), and flavonols (quercetin derivatives) [23,29]. In this study, several compounds not commonly associated with *Euphrasia* species were found in notable concentrations, including the phenolic acid ethyl gallate, the flavanones 7-hydroxyflavanone and 4′-methoxyflavanone, and procyanidin-B2 (Table 1). 

The EO extracts, particularly EOEA and EOB, exhibited relatively high levels of flavonoids and phenolics (Table 1), especially when compared to *E. officinalis* grown in other regions or to other Euphrasia species, such as *E. stricta* [23,30]. Flavonoids and phenolics are well known for their antioxidant and anticholinesterase capacities [31,32,33,34]. As anticipated, antioxidant screening using FRAP and DCFDA assays confirmed that EOEA and EOB possess superior antioxidant activity compared to the other extracts. Furthermore, EODM, which contained the lowest levels of flavonoids and phenolics, demonstrated the weakest antioxidant activity in the DPPH and FRAP assays. This suggests that the high levels of phenolics and flavonoids in EOEA and EOB contribute to these bioactivities. The only inconsistency was observed in the DPPH assay, where EOEA demonstrated a high antioxidant potential as in the other two assays, while EOB showed a relatively lower free radical scavenging capacity. This difference likely reflects varying modes of action between the two extracts, underscoring the importance of using multiple methods to accurately assess the antioxidant potential of an extract [35]. Nevertheless, the enriched presence of compounds with known antioxidant potentials in EOEA and EOB such as ferulic acid, protocatechuic acid, hydroxybenzoic acids, gallic acid, caffeic acid, chlorogenic acid, 7-hydroxyflavanone, quercetin derivatives, apigenin, apigenin-7-*O*-glucoside, and luteolin further supports their antioxidant potential [36,37,38,39,40,41,42,43,44]. Finally, the dose–response relationship observed in the DCFDA assay is consistent with previous findings in antioxidant screenings of other plant extracts [45].

The anticholinesterase potential of *Euphrasia officinalis* has been previously documented in infusions and hydroethanolic extracts [29]. In this study, we have generated extracts with relatively higher anticholinesterase activity, including the EOB in the AChE assay, and the EODM, EOM, EODE, EOEA, and EOB in the BChE assay. This suggests that fractionating plant material into different extracts can enhance its biological activities, revealing properties that otherwise would remain undetected. The anticholinesterase activity observed in these extracts is likely attributed to compounds with documented potential like apigenin, quercetin derivatives, luteolin, caffeic acid, and gallic acid [46,47,48].

The EOW1, EOEA, EODE, EOB, and EOW2 extracts demonstrated a limited yet statistically important anti-neurotoxic effect to Aβ_25-35_-induced neurotoxicity. We have observed more pronounced effects in plant extracts derived from other medicinal plants such as *Sideritis scardica, Salvia fruticosa,* and *Frankenia thymifolia* [45,49,50]. However, this represents the first documented instance of a *Euphrasia* species exhibiting such activity. The notable presence of known neuroprotective molecules such as protocatechuic acid, gallic acid, ethyl gallate, caffeic acid, coumaric acid, ferulic acid, chlorogenic acid, apigenin, apigenin-7-O-glucoside, luteolin, quercetin derivatives such as rutin, myricetin, and procyanidin-B2 in the anti-neurotoxic EO extracts further supports their anti-neurotoxic activity [44,51,52,53,54,55,56,57,58,59,60,61,62,63]. 

The primary compounds identified in these extracts are ferulic acid, apigenin, and quercetin-3-O-rutinoside (rutin). All of these compounds possess antioxidant, anti-inflammatory, and neuroprotective properties, suggesting that they may contribute significantly to the effects observed in the EO extracts. Ferulic acid functions as an antioxidant by scavenging free radicals, enhancing the activity of scavenger enzymes, and inhibiting enzymes that promote free radical formation [36]. It also inhibits AChE activity and reduces the formation of amyloid-beta plaques [64]. Ferulic acid has been reported to attenuate Aβ-induced IL-1β production, thereby reducing neuroinflammation and gliosis. Moreover, it has demonstrated the ability to improve memory deficits in APP/PS1 transgenic mice, a widely used model for Alzheimer’s disease [65]. Apigenin inhibits oxidant enzymes, boosts antioxidant enzyme activity, scavenges free radicals, and acts as a metal chelator [66]. Additionally, apigenin supports acetylcholine restoration in diabetic rats and reduces the accumulation of fibrillar amyloid deposits [67]. Apigenin specifically downregulates Beta-secretase 1 (BACE1), a key enzyme in the amyloidogenic pathway of amyloid precursor protein (APP), as well as β-carboxy-terminal fragment (β-CTF), a cleavage product of the same pathway. Additionally, apigenin has been shown to enhance memory and cognitive functions in APP/PS1 mice [68]. Rutin is a potent free radical scavenger that enhances antioxidant enzyme activity. It has also been shown to reduce cholinesterase activity, inhibit Aβ fibrillization, and mitigate its associated neurotoxicity and mitochondrial dysfunction [55,69,70]. In the Tau-P301S mouse model of tauopathy, rutin has been shown to mitigate Tau hyperphosphorylation, downregulate the NF-κB signaling pathway associated with neuroinflammation and gliosis, restore synaptic integrity in the brain, and improve cognition [71]. Nevertheless, these extracts contain many compounds whose interactions may produce synergistic or additive effects, potentially enhancing the biological properties mentioned above. Conversely, extracts containing neuroprotective, antioxidant, or anticholinesterase substances may not exhibit such effects if antagonistic interactions occur between the compounds [72].

Our findings suggest that *Euphrasia officinalis* L. has the potential to be a natural source of bioactive compounds with therapeutic effects against Alzheimer’s disease. Some of the plant extracts obtained in our study, especially the ethyl acetate and the butanolic ones, exhibited potent antioxidant, AChE, and BChE inhibitory activities, and anti-neurotoxic potential, properties that may be useful against Alzheimer’s disease [73]. These properties can lead to a range of clinical applications. As powerful antioxidants, the extracts may be applicable for protecting neurons from oxidative stress-induced damage, while as cholinesterase inhibitors, they can help maintain higher acetylcholine levels in the brain, which may support cognitive function and delay cognitive decline. Finally, with their anti-neurotoxic activity, EO extracts may also mitigate the harmful effects of Aβ plaques, potentially reducing neurotoxicity associated with AD. 

Conclusively, while the therapeutic potential of these plant extracts is promising, further studies are needed to explore the relevant pharmacological mechanisms in vivo, validate their efficacy, and ensure safety. Nevertheless, EO extracts are another example of natural products that can be employed to develop functional food, dietary supplements, and novel therapeutic agents for disease management.

## 4. Materials and Methods

### 4.1. Chemicals

All the chemicals and solvents were acquired from Sigma-Aldrich (St. Louis, MO, USA), except DMSO purchased from Santa Cruz Biotechnology (Dallas, TX, USA), and the amyloid beta peptides (Aβ_25-35_) obtained from Genscript (Piscataway, NJ, USA). Analytical standards employed were purchased from Extrasynthese (Lyon, France): 4-hydroxybenzoic acid (6099), 3-hydroxybenzoic acid (6098 A), protocatechuic acid (6050), vanillin (6110S), p-hydroxy benzaldehyde (6096), gentisic acid (6048), gallic acid (4993S), ethyl gallate (6446), syringic acid (6011), ferulic acid (4753S), ferulic acid ethyl ester (6245), caffeic acid (6034S), dihydrocaffeic acid (4761 S), trans-cinnamaldehyde (6023), trans-cinnamyl alcohol (6025), m-coumaric acid (6030), p-coumaric acid (4751S), chlorogenic acid (4991S), coumarin (0507S), m-hydroxycoumarin (0505), p-hydroxycoumarin (0517), 7-hydroxycoumarin (0542S), osthol (0541S), eugenol (6178S), isopimpinellin (0536S), xanthotoxin (0530), xanthotoxol (0534), 2′-hydroxyflavanone (1180), 7-hydroxyflavanone (1212), 4′-methoxyflavanone (1185), naringin (1129S), apigenin (1102S), apigenin-7-O-glucoside (1004S), luteolin (1125S), luteolin-7-O-glucoside (1126S), isorhamnetin (120S), quercetin-3-O-rhamnoside (1236S), quercetin-3-O-rutinoside (1139S), quercetin-3-O-galactoside (1027S), myricetin-3-O-galactoside (1355S), myricetin-3-O-rhamnoside (1029S), kaempferol (1124S), kaempferol-3-O-rutinoside (1053), and procyanidin-B2 (0984), and all of them were of >98% purity. Cell culture consumables were purchased from Biosera (Nuaille, France).

### 4.2. Plant Material

The leaves and flowers of *Euphrasia officinalis* L. were gathered from blooming populations in the northern region of Greece (Rhodope Mountains, 41°29′52.1″ N 24°19′01.4″ E) in June 2020. A plant taxonomist identified the plant material. The plant name has been checked with “The World Flora Online” (https://www.worldfloraonline.org) on 16 October 2024 [74]. To ensure optimal preservation, the collected samples were air-dried in a shaded environment, carefully packed in sealed containers, and earmarked for future research. To establish a reference point for future studies, a voucher specimen labeled 0619-Euphroff was preserved in the herbarium of the Laboratory of Pharmacognosy, at the University of Nicosia.

### 4.3. Preparation of EO Extracts 

Fixed-weight (67 g) samples of plant material were placed in a Soxhlet device 0.6 L. The material was defatted with petroleum ether for 27 h, and subjected to exhaustive extraction with dichloromethane and methanol for 49 and 38 h, respectively. The collected fractions (EODM, EOM) were vacuum-evaporated to obtain dry residues. The Soxhlet apparatus temperature was maintained at the boiling point of each solvent during the extraction process.

Next, the plant material was separated using 700 mL of water at 75 °C, and the resulting extract (EOW1) was subsequently dried. The remaining dried methanolic extract was dissolved in 300 mL of hot water (75 °C), filtered, and partitioned consecutively using solvents of increasing polarity (diethyl ether, ethyl acetate, and n-butanol) in the respective proportions of 520 mL [26-fold (20 mL)], 675 mL [45-fold (15 mL)], and 495 mL [33-fold (15 mL)]. Each of the organic layers obtained from each solvent (EODE, EOEA, and EOB) were then concentrated separately under reduced pressure until dryness, while the remaining aqueous extract (EOW2) was also collected (Figure 1). All the extraction and partitioning steps were carried out at room temperature. The extracts were dissolved in DMSO for all assays, except for UPLC-MS/MS analysis, where methanol was used.

### 4.4. Total Phenolic Content (TPC) and Total Flavonoid Content (TFC) Determination

For the determination of the TPC of the extracts, the Folin–Ciocalteu approach was used and measurements were performed in clear 96-well plates [75]. TFC was estimated employing the aluminum chloride colorimetric approach with a few changes to accommodate 96-well plates [76]. 

### 4.5. Ultra-Performance Liquid Chromatography-Tandem Mass Spectrometry (UPLC-MS/MS)

The identification of the various phenolics and flavonoids was conducted with UPLC-MS/MS analysis as previously recorded [77]. Briefly, the chromatographic separation took place on a Waters Acquity UPLC system (Waters Corp., Milford, MA, USA), while a Xevo Triple Quatrable (QqQ) detector (Waters Corp.) was used for the MS/MS analysis. Multiple reaction monitoring (MRM) transitions were used to quantify the analytes. Prior to sample analysis, each standard was carefully adjusted to reach the ideal parameters (cone voltage and collision energy) at 1 ppm concentration (Table S4). Parameters such as linearity, limits of detection (*L*o*D*) and quantification (*L*o*Q*), precision, and accuracy were calculated for each analyte (Table S5). The resultant standard curves for the standards were produced using a linear regression equation utilizing response peak areas as an indicator of the multiple standards’ concentrations, which varied from 0 to 500 ppb (Table S5). With a coefficient of correlation (R^2^) of greater than 0.99, each substance under study demonstrated satisfying linearity (Table S5). Lastly, the analytical method’s repeatability was assessed using the percentage of recovery (Table S5). Five independent experiments were performed. Samples were run under selected ion recording (SIR) mode in order to identify the masses of all the screened compounds using the collision energy obtained from the manual tuning of each of the polyphenols under both positive and negative electrospray (ESI±) ionization (Figure S1). 

### 4.6. DPPH Radical Scavenging Activity Assay

The stable radical 2,2-diphenyl-1-picrylhydrazyl (DPPH•) was employed in the DPPH• test to measure the antioxidant activity, which was carried out in agreement with Parejo et al.’s technique with minor adjustments [78]. Various extract concentrations were generated in order to gauge the antioxidant capacity of all seven extracts. In a nutshell, an aliquot of diluted extract was dissolved in a 1:40 ratio in the mother DPPH• solution (0.02 mM), after which the combination was vortexed. The reaction mixture was then stored at RT. Absorbance at 517 nm was measured at different time intervals using a spectrophotometer. The reaction’s plateau was identified by a drop in absorbance, which was then measured. The absorbance of the extracts without DPPH• was subtracted from the absorbance with DPPH•. A calibration curve was employed to determine the concentration of DPPH• in the reaction medium. For each extract concentration investigated, the percentage of DPPH• remaining at steady state was determined as follows: percentage of residual DPPH• is calculated as [DPPH•] at t = T/[DPPH•] at t = 0, where T is the time required to achieve the steady state. The amount of extract needed to decrease the starting DPPH• concentration by 50% (EC_50_) was used to represent each fraction’s antioxidant capacity. The antiradical efficiency (AE) is computed as AE = 1/EC_50_.

### 4.7. Ferric Reducing Antioxidant Power (FRAP) Assay 

The Benzie et al. [79] approach was used to carry out the FRAP test. A 200 μL appropriately diluted aliquot of each plant extract was added to 1800 μL of freshly prepared FRAP solution that was formed of 3.75 mL of TPTZ (0.01 M) in 40 mM HCl, 3.75 mL of iron(III) chloride solution (0.02 M), and 37.5 mL of an acetate buffer (0.03 M, pH 3.6). The solution remained at RT for 10 min before the absorbance was measured at 593 nm. The antioxidant activity was represented as ascorbic acid equivalents (AAE mol/g sample) and Trolox equivalent antioxidant capacity (TEAC mol/g sample), based on corresponding standard curves.

### 4.8. Cell Culture 

SH-SY5Y human neuroblastoma cells were purchased by DSMZ (Braunschweig, Germany) and were routinely cultured in Dulbecco’s Modified Eagle Medium including 10% fetal bovine serum, 5% horse serum, 1% antibiotics (penicillin and streptomycin), and preserved at 37 °C and 5% CO_2_.

### 4.9. Intracellular Oxidative Stress Evaluation 

To evaluate the extracts’ ability to counteract intracellular oxidative strays, SH-SY5Y cells (1.5 × 10^4^/well) were seeded in a black 96-well plate, and 24 h later various concentrations of the extracts were added along with 50 μΜ hydrogen peroxide for 1 h. DCF-DA assay was then employed to evaluate the presence of radical oxygen species [80]. Trolox served as a standard antioxidant, and three independent tests were conducted. 

### 4.10. Acetylcholinesterase (AChE) and Butyrylcholinesterase (BChE) Activity Inhibitory Assay

Initially, AChE and BChE were dissolved in 50 mM Tris-HCl pH 8 supplemented with 0.1% *w/v* bovine serum albumin at a concentration of 5 U/mL. 5,5′-Dithiobis(2-nitrobenzoic acid) (DTNB) was dissolved in 50 mM Tris-HCl pH 8 containing 0.1 M NaCl and 0.02 M MgCl_2_ at a 3 mM concentration, and acetylthiocholine iodide (ATCI) and S-butyrylthiocholine chloride (BTC) were dissolved in 50 mM Tris-HCl pH 8 at a 1.5 mM concentration just before use.

To evaluate the AChE or BChE inhibitory activity of the extracts, clear 96-well plates were used. In each well, 5 μL of various concentrations of the extracts dissolved in DMSO were mixed with 88.5 μL of 50 mM Tris-HCl pH 8 and 1.5 μL of 5 U/mL AChE or BChE. In the control wells, the vehicle solvent was added instead of extracts, and in the blank control wells 50 mM Tris-HCl pH 8 replaced all other reagents. In the positive control wells, various concentrations of donepezil were added instead of extracts. The plate was then shaken for 15 min at RT. Next, 125 μL of 3 mM DTNB and 30 μL of 1.5 mM ATCI or BTC were added to each well, and after ten seconds of shaking, absorbance was measured at 0 and 10 min. The absorbance was read at 405 nm in a microplate reader, using the following formula after subtracting the blank control readings from all measurements:

$$\%\mathrm{AChE}\;\mathrm{or}\;\mathrm{BChE}\;\mathrm{inhibitory}\;\mathrm{activity}=\frac{\left(\Delta A\;\mathrm{Solvent}\;\mathrm{control}-\Delta A\;\mathrm{Sample}\right)}{\Delta A\;\mathrm{Solvent}\;\mathrm{control}}$$

- ∆A = absorbance of a sample or a solvent control well at t = 10 min minus absorbance at t = 0.

Assays were conducted in triplicates, and at least three independent experiments were performed.

### 4.11. Peptides Preparation

Aβ_25-35_ peptides were prepared in sterile distilled water at a concentration of 1 mM and maintained at 37 °C for 7 days to form aggregates. Aβ_25-35_ was stored in aliquots in the freezer until use.

### 4.12. MTT Cell Viability Assay 

SH-SY5Y cells underwent an MTT assay to examine SH-SY5Y cell viability following incubation with EO extracts and/or Aβ_25-35_ aggregates [81]. A quantity of 3 × 10^4^ cells was plated per well in clear 96-well plates, and the next day treated with various concentrations of the extracts and/or 30 μΜ of Aβ_25-35_ aggregates for 48 h. Then, 45 μg/mL thiazolyl blue tetrazolium bromide was introduced, followed by incubation for 4 h at 37 °C. Afterward, 150 μL DMSO was included to dissolve the formazan crystals. After shaking for half an hour, the optical density was measured at 570 nm. Cell viability was computed as the ratio of the optical density of the treated cells to control cells, after subtraction of the blank reading. At least four independent experiments were performed.

### 4.13. Statistics

The data are displayed in the form of means ± standard deviation (SD) or standard error of the mean (SEM). Statistical importance for comparing control with the cells under treatment was performed by one-way analysis of variance (ANOVA) followed by Dunnett’s post-hoc test. Similarly, to compare various conditions, one-way ANOVA followed by Tukey’s test was performed. The above data analyses were conducted using the statistical software GraphPad Prism v. 9.3.1 (GraphPad Software, CA, United States). The threshold for statistical significance was set at *p* < 0.05.

## 5. Conclusions

This work is the first to describe the neuroprotective potential of a *Euphrasia* species, specifically *Euphrasia officinalis* L. Extracts derived from this plant are rich in phenolics and flavonoids, demonstrating antioxidant, anticholinesterase, and anti-neurotoxic properties. Among the extracts tested, the ethyl acetate and butanolic extracts were found to be the most effective in these areas. These findings suggest that these extracts could be valuable in the development of health-beneficial functional food, supplements, and medications.

## Figures and Tables

**Table 1:** Quantitative data displaying the phytochemical composition of the seven *E. officinalis* fractions: dichloromethane, methanol, water (1), diethyl ether, ethyl acetate, butanol, water (2). Data collections were obtained via UPLC-ESI(±)-QqQ (UPLC-(electrospray ionization-triple quadrupole)) and standardized at two decimal places. The data represent means ± standard deviation (SD) of five independent studies. Means and SDs preceded by distinct letters in a category significantly vary, as indicated by Tukey’s test (*p* < 0.05). The initials n.d. represent data for which the associated signals were not detectable.

EXTRACT	EODM	EOM	EOW1	EODE	EOEA	EOB	EOW2
Benzoic acid derivatives (μg/g of dry extract)							
m-hydroxy benzoic acid	n.d.	1.25 ± 0.10 ^a^	1.34 ± 0.02 ^a^	6.12 ± 0.54 ^b^	45.55 ± 2.32 ^d^	24.32 ± 0.99 ^c^	n.d.
p-hydroxy benzoic acid	1.32 ± 0.01 ^a^	6.16 ± 0.23 ^c^	n.d.	n.d.	64.54 ± 3.14 ^e^	40.43 ± 2.21 ^d^	3.45 ± 0.15 ^b^
Protocatechuic acid	n.d.	4.44 ± 1.23 ^a^	4.21 ± 0.29 ^a^	3.33 ± 0.12 ^a^	112.36 ± 9.87 ^c^	55.48 ± 2.45 ^b^	n.d.
Vanillin	n.d.	0.60 ± 0.01 ^b^	0.26 ± 0.01 ^a^	0.21 ± 0.01 ^a^	11.65 ± 0.22 ^c^	12.32 ± 1.03 ^c^	n.d.
Gentisic acid	n.d.	1.84 ± 0.04 ^b^	4.43 ± 0.22 ^d^	3.41 ± 0.22 ^c^	8.45 ± 0.46 ^e^	n.d.	0.55 ± 0.03 ^a^
Gallic acid derivatives (μg/g of dry extract)							
Gallic acid	0.95 ± 0.56 ^a^	86.36 ± 2.15 ^f^	6.45 ± 0.03 ^c^	3.21 ± 0.01 ^b^	245.65 ± 2.14 ^g^	64.14 ± 1.21 ^e^	11.48 ± 0.89 ^d^
Ethyl gallate	n.d.	24.13 ± 1.03 ^d^	12.12 ± 0.10 ^c^	6.08 ± 0.24 ^b^	149.56 ± 4.13 ^f^	60.15 ± 1.98 ^e^	0.96 ± 0.01 ^a^
Syringic acid	n.d.	3.94 ± 0.13 ^a^	n.d.	n.d.	47.31 ± 2.19 ^c^	9.98 ± 2.14 ^b^	4.96 ± 0.36 ^a^
Cinnamic acid derivatives (μg/g of dry extract)							
Ferulic acid	4.89 ± 0.17 ^b^	35.65 ± 2.36 ^d^	3.60 ± 0.13 ^a^	20.21 ± 1.74 ^c^	978.98 ± 12.65 ^f^	645.55 ± 11.11 ^e^	n.d.
Ferulic acid ethyl ester	0.84 ± 0.01 ^a^	3.65 ± 0.14 ^c^	n.d.	2.68 ± 0.02 ^b^	64.18 ± 2.94 ^e^	25.12 ± 1.48 ^d^	n.d.
Caffeic acid	5.49 ± 0.23 ^b^	13.62 ± 0.97 ^d^	14.18 ± 0.06 ^d^	10.98 ± 0.02 ^c^	179.92 ± 11.11 ^f^	100.98 ± 2.32 ^e^	1.14 ± 0.01 ^a^
Dihydrocaffeic acid	n.d.	41.10 ± 2.61 ^c^	n.d.	26.87 ± 1.12 ^b^	348.98 ± 20.07 ^e^	73.18 ± 6.01 ^d^	2.14 ± 0.01 ^a^
Trans-cinnamaldehyde	n.d.	4.87 ± 0.12 ^a^	n.d.	5.67 ± 0.48 ^a^	21.55 ± 1.78 ^c^	10.21 ± 0.10 ^b^	n.d.
Trans-cinnamyl alcohol	0.21 ± 0.01 ^a^	n.d.	0.48 ± 0.01 ^b^	0.22 ± 0.01 ^a^	n.d.	6.54 ± 0.01 ^c^	n.d.
m-coumaric acid	n.d.	8.90 ± 0.32 ^b^	n.d.	5.51 ± 0.26 ^a^	34.98 ± 1.40 ^d^	12.38 ± 1.01 ^c^	n.d.
p-coumaric acid	n.d.	7.78 ± 0.28 ^b^	n.d.	6.18 ± 0.36 ^a^	n.d.	5.24 ± 0.24 ^a^	n.d.
Chlorogenic acid	4.11 ± 0.23 ^b^	41.21 ± 0.94 ^f^	7.18 ± 0.36 ^c^	21.53 ± 1.47 ^e^	148.98 ± 10.41 ^g^	14.46 ± 0.11 ^d^	2.14 ± 0.02 ^a^
Coumarin derivatives (μg/g of dry extract)							
Coumarin	2.12 ± 0.01 ^b^	6.48 ± 0.51 ^c^	19.26 ± 1.29 ^d^	5.13 ± 0.27 ^c^	22.13 ± 0.61 ^d^	n.d.	1.11 ± 0.01 ^a^
m-hydroxycoumarin	n.d.	n.d.	10.95 ± 0.31 ^b^	n.d.	12.48 ± 0.58 ^c^	9.00 ± 0.04 ^b^	2.14 ± 0.01 ^a^
p-hydroxycoumarin	9.19 ± 0.67 ^b^	n.d.	n.d.	n.d.	n.d.	5.11 ± 0.02 ^a^	n.d.
7- hydroxycoumarin	1.86 ± 0.07 ^a^	14.04 ± 1.02 ^d^	12.01 ± 0.13 ^d^	8.42 ± 0.26 ^b^	13.32 ± 1.04 ^d^	10.48 ± 1.00 ^c^	n.d.
Osthol	6.12 ± 0.13 ^d^	n.d.	n.d.	2.21 ± 0.01 ^c^	n.d.	1.11 ± 0.01 ^b^	0.69 ± 0.01 ^a^
Phenolic derivatives (μg/g of dry extract)							
Eugenol	n.d.	1.40 ± 0.01 ^b^	n.d.	0.48 ± 0.01 ^a^	5.14 ± 0.14 ^c^	1.21 ± 0.10 ^b^	n.d.
Total Phenolic Content (TPC) (mg of gallic acid eq/g of extract)							
	22.51 ± 1.01 ^a^	86.56 ± 3.54 ^c^	91.64 ± 3.78 ^c^	98.10 ± 2.01 ^d^	405.98 ± 16.54 ^f^	236.89 ± 12.68 ^e^	47.13 ± 1.54 ^b^
Furanocoumarin derivatives (μg/g of dry extract)							
Isopimpinellin	n.d.	0.58 ± 0.02 ^a^	n.d.	n.d.	4.12 ± 0.32 ^b^	n.d.	n.d.
Xanthotoxin	0.21 ± 0.01 ^a^	0.92 ± 0.03 ^b^	n.d.	n.d.	2.98 ± 0.24 ^d^	1.48 ± 0.08 ^c^	n.d.
Xanthotoxol	0.11 ± 0.01 ^a^	n.d.	n.d.	n.d.	12.98 ± 1.03 ^c^	5.33 ± 0.17 ^b^	n.d.
Flavanone derivatives (μg/g of dry extract)							
2′-hydroxyflavanone	n.d.	n.d.	n.d.	6.45 ± 0.45 ^a^	45.68 ± 3.05 ^c^	24.12 ± 1.58 ^b^	5.38 ± 0.13 ^a^
7-hydroxyflavanone	1.44 ± 0.01 ^a^	3.00 ± 0.14 ^b^	n.d.	14.48 ± 1.21 ^d^	678.18 ± 26.18 ^f^	357.98 ± 15.93 ^e^	10.42 ± 0.70 ^c^
4′-methoxyflavanone	n.d.	n.d.	n.d.	26.26 ± 2.02 ^a^	345.16 ± 19.45 ^c^	197.64 ± 11.99 ^b^	n.d.
Naringin	n.d.	2.96 ± 0.11 ^b^	1.89 ± 0.03 ^a^	3.24 ± 0.11 ^b^	8.35 ± 0.61 ^d^	4.21 ± 0.36 ^b, c^	n.d.
Flavone derivatives (μg/g of dry extract)							
Apigenin	4.62 ± 0.26 ^b^	12.28 ± 0.13 ^c^	n.d.	348.15 ± 15.48 ^d^	897.48 ± 36.24 ^f^	657.98 ± 27.13 ^e^	3.21 ± 0.24 ^a^
Apigenin-7-O-glucoside	n.d.	n.d.	n.d.	210.69 ± 15.97 ^a^	743.14 ± 17.48 ^c^	555.47 ± 15.87 ^b^	n.d.
Luteolin	n.d.	n.d.	0.22 ± 0.01 ^a^	73.85 ± 6.12 ^b^	169.18 ± 12.12 ^d^	97.15 ± 3.37 ^c^	n.d.
Luteolin-7-O-glucoside	1.82 ± 0.11 ^a^	8.53 ± 0.69 ^c^	3.77 ± 0.13 ^b^	16.98 ± 1.21 ^d^	49.98 ± 3.15 ^e^	14.69 ± 1.21 ^d^	n.d.
Flavonol derivatives (μg/g of dry extract)							
Isorhamnetin	2.64 ± 0.02 ^a^	2.65 ± 0.10 a	n.d.	2.54 ± 0.11 a	67.98 ± 5.50 c	41.24 ± 2.40 b	n.d.
Quercetin-3-O-rhamnoside	n.d.	0.64 ± 0.02 ^a^	n.d.	n.d.	456.13 ± 13.44 ^c^	98.45 ± 6.47 ^b^	n.d.
Quercetin-3-O-rutinoside	n.d.	n.d.	0.68 ± 0.03 ^a^	n.d.	947.98 ± 46.98 ^c^	236.79 ± 14.44 ^b^	n.d.
Quercetin-3-O-galactoside	n.d.	n.d.	0.020 ± 0.001 ^a^	n.d.	6.54 ± 0.21 ^c^	0.98 ± 0.01 ^b^	n.d.
Myricetin-3-O-galactoside	1.22 ± 0.01 ^a^	2.13 ± 0.01 ^b^	n.d.	2.01 ± 0.10 ^b^	183.31 ± 10.18 ^e^	54.48 ± 2.34 ^d^	3.21 ± 0.24 ^c^
Myricetin-3-O-rhamnoside	0.16 ± 0.01 ^a^	0.20 ± 0.01 ^a^	1.47 ± 0.01 ^b^	n.d.	16.65 ± 0.24 ^d^	10.98 ± 0.10 ^c^	1.00 ± 0.001 ^b^
Kaempferol	n.d.	1.29 ± 0.03 ^a^	n.d.	n.d.	54.98 ± 3.69 ^c^	21.21 ± 1.53 ^b^	n.d.
Kaempferol-3-O-rutinoside	n.d.	11.48 ± 0.41 ^c^	n.d.	9.87 ± 0.48 ^b^	96.12 ± 4.56 ^d^	n.d.	4.98 ± 0.08 ^a^
Procyanidins (μg/g of dry extract)							
Procyanidin-B2	0.21 ± 0.01 ^a^	n.d.	44.65 ± 2.31 ^d^	n.d.	5.65 ± 0.12 ^b^	n.d.	10.48 ± 1.06 ^c^
Total Flavonoid Content (TFC) (μmol catechin eq/g of extract)							
	24.65 ± 1.23 ^a^	548.26 ± 2.45 ^e^	136.66 ± 6.15 ^c^	230.56 ± 7.98 ^d^	766.98 ± 10.13 ^g^	651.33 ± 12.21 ^f^	82.11 ± 2.17 ^b^
Total Flavonoid Content (TFC) (μmol rutin eq/g of extract)							
	20.15 ± 1.11 ^a^	845.37 ± 9.87 ^e^	187.98 ± 8.88 ^c^	285.47 ± 16.69 ^d^	988.12 ± 13.32 ^f^	979.98 ± 14.54 ^f^	91.52 ± 6.14 ^b^

**Table 2:** The antioxidant capacity of the seven EO extracts as assessed through DPPH•, FRAP, and DCFDA assays. The results are shown as averages ± standard deviation followed by different letters that indicate statistical significance within the same row.

		EODM	EOM	EOW1	EODE	EOEA	EOB	EOW2
DPPH•	EC_50_ (mg extract/mg DPPH•)	14.18 ± 1.91 ^b^	0.42 ± 0.01 ^a^	0.39 ± 0.02 ^a^	0.50 ± 0.02 ^a^	0.23 ± 0.01 ^a^	0.73 ± 0.04 ^a^	0.66 ± 0.03 ^a^
	AE	0.07	2.36	2.59	2.00	4.43	1.38	1.51
FRAP	μmol AAE/g	57.18 ± 3.67 ^a^	724.44 ± 18.76 ^d^	364.29 ± 3.38 ^bc^	439.82 ± 9.14 ^c^	2992.48 ± 256.74 ^f^	1192.21 ± 41.66 ^e^	106.44 ± 17.84 ^ab^
	μmol TEAC/g	73.72 ± 6.37 ^a^	793.92 ± 25.68 ^c^	528.34 ± 42.99 ^abc^	604.38 ± 52.65 ^bc^	3658.90 ± 430.45 ^e^	1523.40 ± 125.42 ^d^	171.80 ± 7.04 ^ab^
DCFDA	IC_50_ (μg/mL)	16.05 ± 3.61 ^b^	21.00 ± 7.89 ^bc^	33.34 ± 5.18 ^c^	27.37 ± 1.83 ^bc^	2.80 ± 2.28 ^a^	3.38 ± 2.26 ^a^	31.28 ± 5.16 ^c^

**Table 3:** Cholinesterase inhibitory activity of EO extracts. The results are shown as averages ± standard error of the mean followed by different letters that indicate statistical significance within the same row. The inhibition values were taken at 1 mg/mL for the EO extracts, and at 32 ng/mL (AChE) and 4 μg/mL (BChE) for the positive control Donepezil that was excluded from statistical analysis. n.d. for non-detected.

		EODM	EOM	EOW1	EODE	EOEA	EOB	EOW2	Donepezil
AChE	IC_25_ (μg/mL)	760.70 ± 39.02 ^bc^	848.19 ± 69.21 ^c^	>1000	>1000	542.42 ± 71.41 ^b^	274.29 ± 14.56 ^a^	>1000	4.17 ± 0.16 ng/mL
	IC_50_ (μg/mL)	>1000	>1000	>1000	>1000	>1000	646.93 ± 33.03	>1000	10.16 ± 0.16 ng/mL
	Inhibition %	33.27 ± 5.83 ^bc^	34.15 ± 4.34 ^bc^	11.07 ± 3.73 ^a^	23.27 ± 3.50 ^ab^	49.70 ± 2.46 ^c^	66.93 ± 1.42 ^d^	n.d.	76.16 ± 0.51
BChE	IC_50_ (μg/mL)	267.82 ± 45.70 ^a^	615.95 ± 101.86 ^b^	>1000	315.75 ± 62.21 ^a^	276.79 ± 41.74 ^a^	288.02 ± 33.19 ^a^	>1000	1.26 ± 0.03
	Inhibition %	72.48 ± 0.84 ^cd^	63.81 ± 3.92 ^c^	4.28 ± 1.12 ^a^	34.96 ± 8.81 ^b^	87.13 ± 6.46 ^de^	97.58 ± 2.42 ^e^	7.56 ± 2.43 ^a^	84.09 ± 1.55

## Data Availability

The data are contained within the article.

## Supplementary Materials

The following supporting information can be downloaded at: https://www.mdpi.com/article/10.3390/ijms252312902/s1.
