# Supplementary material for: Chemical Characterization and Assessment of the Neuroprotective Potential of Euphrasia officinalis
Source: Int J Mol Sci. 2024 Nov 30;25(23):12902. doi: 10.3390/ijms252312902 (PMC11641456; doi:10.3390/ijms252312902)
Supplement: Supplementary file 1 [file ijms-25-12902-s001.zip › ijms-3297896-supplementary.pdf]

Chemical characterization and assessment of the neuroprotective potential of *Euphrasia officinalis*

SUPPLEMENTARY MATERIAL

Antonis Ververis [1], Sotiris Kyriakou [2], Hariklia Paraskeva [1], Mihalis I. Panayiotidis [2], Michael Plioukas [3] and Kyproula Christodoulou [1,*]

[1] Neurogenetics Department, The Cyprus Institute of Neurology and Genetics, Nicosia 2371, Cyprus; antonisv@cing.ac.cy (A.V.); charicleaparaskeva@gmail.com (H.P.)
[2] Department of Cancer Genetics, Therapeutics and Ultrastructural Pathology, The Cyprus Institute of Neurology and Genetics, Nicosia 2371, Cyprus; sotirisk@cing.ac.cy (S.K.); mihalisp@cing.ac.cy (M.I.P.)
[3] Department of Life and Health Sciences, School of Sciences and Engineering, University of Nicosia, Nicosia 2417, Cyprus; pmichael.gr@hotmail.com
* Correspondence: roula@cing.ac.cy; Tel.: +357-22-392649

Table S1: Dunnett's multiple comparisons test conducted for the DCF-DA assay. The presence of cellular ROS in control cells treated with 50 μM H_2O_2 was compared with the presence of cellular ROS in cells treated with various concentrations of each extract and 50 μM H_2O_2. *** refers to $p < 0.001$ statistical significance, compared with control cells only treated with 50 μm H_2O_2.

**Dunnett's multiple comparisons test**	**Mean 1**	**Mean 2**	**Mean Diff.**	**95.00% CI of diff.**	**Below threshold?**	**Summary**	**Adjusted P Value**
Control vs. Untreated	100	11.63	88.37	58.57 to 118.2	Yes	***	<.001
Control vs. 500 μM Trolox	100	15.14	84.86	55.07 to 114.7	Yes	***	<.001
Control vs. EODM 200 μg/mL	100	18.82	78.41	48.61 to 108.2	Yes	***	<.001
Control vs. EODM 100 μg/mL	100	25.17	70.01	40.21 to 99.80	Yes	***	<.001
Control vs. EODM 50 μg/mL	100	33.81	57.9	28.10 to 87.69	Yes	***	<.001
Control vs. EODM 2 μg/mL	100	58.23	37.06	7.267 to 66.85	Yes	**	0.005
Control vs. EOM 200 μg/mL	100	22.63	77.49	47.69 to 107.3	Yes	***	<.001
Control vs. EOM 100 μg/mL	100	22.26	79.91	50.11 to 109.7	Yes	***	<.001
Control vs. EOM 50 μg/mL	100	25.03	80.83	51.04 to 110.6	Yes	***	<.001
Control vs. EOM 2 μg/mL	100	77.09	45.34	15.55 to 75.14	Yes	***	<.001
Control vs. EOW1 200 μg/mL	100	27.99	75.19	45.39 to 105.0	Yes	***	<.001
Control vs. EOW1 100 μg/mL	100	31.61	75.74	45.95 to 105.5	Yes	***	<.001
Control vs. EOW1 50 μg/mL	100	35.5	70.71	40.92 to 100.5	Yes	***	<.001
Control vs. EOW1 2 μg/mL	100	93.71	29.12	-0.6783 to 58.91	No	ns	0.061
Control vs. EODE 200 μg/mL	100	21.34	81.56	51.76 to 111.4	Yes	***	<.001
Control vs. EODE 100 μg/mL	100	24.06	81.09	51.29 to 110.9	Yes	***	<.001
Control vs. EODE 50 μg/mL	100	29.45	78.63	48.83 to 108.4	Yes	***	<.001
Control vs. EODE 2 μg/mL	100	87.32	36.12	6.328 to 65.92	Yes	**	0.007
Control vs. EOEA 200 μg/mL	100	29.12	62.36	32.56 to 92.15	Yes	***	<.001
Control vs. EOEA 100 μg/mL	100	17.24	75.12	45.33 to 104.9	Yes	***	<.001
Control vs. EOEA 50 μg/mL	100	13.78	80.95	51.16 to 110.7	Yes	***	<.001
Control vs. EOEA 2 μg/mL	100	25.4	65.47	35.67 to 95.26	Yes	***	<.001
Control vs. EOEA 1 μg/mL	100	39.96	60.04	23.55 to 96.53	Yes	***	<.001
Control vs. EOEA 0.1 μg/mL	100	92.89	7.107	-29.38 to 43.60	No	ns	>.999
Control vs. EOB 200 μg/mL	100	18.74	79.97	50.18 to 109.8	Yes	***	<.001

Control vs. EOB 100 µg/mL	100	16.41	81.49	51.69 to 111.3	Yes	***	<.001
Control vs. EOB 50 µg/mL	100	13.83	83.41	53.62 to 113.2	Yes	***	<.001
Control vs. EOB 2 µg/mL	100	22.49	61.15	31.36 to 90.95	Yes	***	<.001
Control vs. EOB 1 µg/mL	100	50.22	49.78	13.29 to 86.27	Yes	**	0.001
Control vs. EOB 0.1 µg/mL	100	87.51	12.49	-24.00 to 48.98	No	ns	0.993
Control vs. EOW2 200 µg/mL	100	30.01	72.48	42.69 to 102.3	Yes	***	<.001
Control vs. EOW2 100 µg/mL	100	31.28	64.31	34.51 to 94.10	Yes	***	<.001
Control vs. EOW2 50 µg/mL	100	32.75	58.14	28.35 to 87.94	Yes	***	<.001
Control vs. EOW2 2 µg/mL	100	100.2	-9.852	-39.65 to 19.94	No	ns	0.994

Table S2: Dunnett's multiple comparisons test for the cytotoxicity assay of the extracts. The viability of untreated control cells was compared to that of cells exposed to varying concentrations of each extract. A ** indicates $p < 0.01$, while *** denotes $p < 0.001$, showing statistical significance in comparison to untreated control cells.

Dunnett's multiple comparisons test	Mean 1	Mean 2	Mean Diff.	95.00% CI of diff.	Below threshold?	Summary	Adjusted P Value
Control vs. EODM 200 µg/mL	100	53.45	46.56	21.51 to 71.60	Yes	***	<.001
Control vs. EODM 100 µg/mL	100	86.61	13.4	-11.65 to 38.44	No	ns	0.726
Control vs. EODM 50 µg/mL	100	90.37	9.635	-15.41 to 34.68	No	ns	0.97
Control vs. EOM 400 µg/mL	100	14.24	85.76	60.72 to 110.8	Yes	***	<.001
Control vs. EOM 200 µg/mL	100	96.6	3.405	-21.64 to 28.45	No	ns	>.999
Control vs. EOM 100 µg/mL	100	90.51	9.493	-15.55 to 34.54	No	ns	0.973
Control vs. EOM 50 µg/mL	100	89.04	10.97	-14.08 to 36.01	No	ns	0.913
Control vs. EOW1 200 µg/mL	100	71.4	28.61	3.558 to 53.65	Yes	*	0.015
Control vs. EOW1 100 µg/mL	100	78.72	21.28	-3.764 to 46.33	No	ns	0.149
Control vs. EOW1 50 µg/mL	100	80.37	19.63	-5.417 to 44.68	No	ns	0.225
Control vs. EODE 200 µg/mL	100	60.73	39.28	14.23 to 64.32	Yes	***	<.001
Control vs. EODE 100 µg/mL	100	78.31	21.69	-3.359 to 46.73	No	ns	0.133
Control vs. EODE 50 µg/mL	100	76.83	23.17	-1.879 to 48.21	No	ns	0.088
Control vs. EOEA 200 µg/mL	100	70.46	29.54	4.493 to 54.59	Yes	*	0.011
Control vs. EOEA 100 µg/mL	100	87.19	12.81	-12.24 to 37.86	No	ns	0.778
Control vs. EOEA 50 µg/mL	100	93.12	6.88	-18.17 to 31.93	No	ns	0.995
Control vs. EOB 400 µg/mL	100	9.588	90.41	65.37 to 115.5	Yes	***	<.001
Control vs. EOB 200 µg/mL	100	75.95	24.05	-0.9995 to 49.09	No	ns	0.068
Control vs. EOB 100 µg/mL	100	85.92	14.09	-10.96 to 39.13	No	ns	0.662
Control vs. EOB 50 µg/mL	100	95.97	4.03	-21.02 to 29.08	No	ns	>.999
Control vs. EOW2 400 µg/mL	100	58.08	41.93	16.88 to 66.97	Yes	***	<.001
Control vs. EOW2 200 µg/mL	100	104.9	-4.905	-29.95 to 20.14	No	ns	>.999
Control vs. EOW2 100 µg/mL	100	103.2	-3.175	-28.22 to 21.87	No	ns	>.999
Control vs. EOW2 50 µg/mL	100	102	-1.975	-27.02 to 23.07	No	ns	>.999

Table S3: Dunnett's multiple comparisons test for the anti-neurotoxic assay of the extracts. The viability of cells treated solely with 30 μM A$\beta_{25\text{-}35}$ was compared to that of cells exposed to various concentrations of each extract in combination with 30 μM A$\beta_{25\text{-}35}$. A * denotes statistical significance at $p < 0.05$, while ** indicates $p < 0.01$, relative to cells treated only with 30 μM A$\beta_{25\text{-}35}$.

Dunnett's multiple comparisons test	Mean 1	Mean 2	Mean Diff.	95.00% CI of diff.	Below threshold?	Summary	Adjusted P Value
30 μM Aβ25-35 vs. EODM 100 μg/mL	50.18	42.42	7.766	-0.8694 to 16.40	No	ns	0.11
30 μM Aβ25-35 vs. EODM 50 μg/mL	50.18	51.69	-1.505	-10.14 to 7.131	No	ns	>.999
30 μM Aβ25-35 vs. EODM 20 μg/mL	50.18	55.65	-5.466	-14.10 to 3.170	No	ns	0.534
30 μM Aβ25-35 vs. EODM 2 μg/mL	50.18	55.27	-5.089	-13.72 to 3.547	No	ns	0.64
30 μM Aβ25-35 vs. EOM 200 μg/mL	50.18	46.33	3.855	-4.781 to 12.49	No	ns	0.933
30 μM Aβ25-35 vs. EOM 100 μg/mL	50.18	46.75	3.431	-5.205 to 12.07	No	ns	0.979
30 μM Aβ25-35 vs. EOM 50 μg/mL	50.18	49.29	0.8983	-7.738 to 9.534	No	ns	>.999
30 μM Aβ25-35 vs. EOM 20 μg/mL	50.18	54.16	-3.981	-12.62 to 4.655	No	ns	0.913
30 μM Aβ25-35 vs. EOM 2 μg/mL	50.18	49.75	0.4386	-8.197 to 9.074	No	ns	>.999
30 μM Aβ25-35 vs. EOW1 100 μg/mL	50.18	47.85	2.338	-6.298 to 10.97	No	ns	0.999
30 μM Aβ25-35 vs. EOW1 50 μg/mL	50.18	47.35	2.834	-5.801 to 11.47	No	ns	0.994
30 μM Aβ25-35 vs. EOW1 20 μg/mL	50.18	60.35	-10.17	-18.80 to -1.531	Yes	**	0.01
30 μM Aβ25-35 vs. EOW1 2 μg/mL	50.18	58.91	-8.726	-17.36 to -0.09017	Yes	*	0.046
30 μM Aβ25-35 vs. EODE 100 μg/mL	50.18	46.67	3.515	-5.121 to 12.15	No	ns	0.972
30 μM Aβ25-35 vs. EODE 50 μg/mL	50.18	51.68	-1.491	-10.13 to 7.144	No	ns	>.999
30 μM Aβ25-35 vs. EODE 20 μg/mL	50.18	59.54	-9.356	-17.99 to -0.7205	Yes	*	0.024
30 μM Aβ25-35 vs. EODE 2 μg/mL	50.18	55.73	-5.545	-14.18 to 3.090	No	ns	0.513
30 μM Aβ25-35 vs. EOEA 100 μg/mL	50.18	48.46	1.724	-6.912 to 10.36	No	ns	>.999
30 μM Aβ25-35 vs. EOEA 50 μg/mL	50.18	55.69	-5.506	-14.14 to 3.130	No	ns	0.523
30 μM Aβ25-35 vs. EOEA 20 μg/mL	50.18	56.8	-6.62	-15.26 to 2.016	No	ns	0.265
30 μM Aβ25-35 vs. EOEA 2 μg/mL	50.18	59.03	-8.846	-17.48 to -0.2106	Yes	*	0.041
30 μM Aβ25-35 vs. EOB 200 μg/mL	50.18	44.4	5.78	-2.856 to 14.42	No	ns	0.451
30 μM Aβ25-35 vs. EOB 100 μg/mL	50.18	48.74	1.445	-7.191 to 10.08	No	ns	>.999
30 μM Aβ25-35 vs. EOB 50 μg/mL	50.18	52.06	-1.878	-10.51 to 6.758	No	ns	>.999
30 μM Aβ25-35 vs. EOB 20 μg/mL	50.18	60.22	-10.04	-18.67 to -1.400	Yes	*	0.012

30 µM Aβ25-35 vs. EOB 2 µg/mL	50.18	56.09	-5.91	-14.55 to 2.726	No	ns	0.418
30 µM Aβ25-35 vs. EOW2 200 µg/mL	50.18	56.65	-6.464	-15.10 to 2.172	No	ns	0.295
30 µM Aβ25-35 vs. EOW2 100 µg/mL	50.18	56.82	-6.634	-15.27 to 2.002	No	ns	0.263
30 µM Aβ25-35 vs. EOW2 50 µg/mL	50.18	57.98	-7.799	-16.43 to 0.8372	No	ns	0.107
30 µM Aβ25-35 vs. EOW2 20 µg/mL	50.18	61.54	-11.36	-19.99 to -2.723	Yes	**	0.002
30 µM Aβ25-35 vs. EOW2 2 µg/mL	50.18	58.44	-8.259	-16.90 to 0.3765	No	ns	0.071

Table S4: Multiple Reaction Monitoring (MRM) conditions for polyphenolic acids and flavonoids used for content quantification in *Euphrasia officinalis* via UPLC-MS/MS analysis.

Polyphenolic compound	Chemical formula	Molecular weight (g/mol)	$[M-H]^{\pm}$ (m/z)	ESI mode	MS^2 fragments (m/z)	Cone voltage (V)	Collision energy (eV)	Retention time (R_t)
				**Benzoic acid derivatives**				
m-hydroxy benzoic acid	$C_7H_6O_3$	138.12	137.05	-	92.9	22	10	2.34
p-hydroxy benzoic acid	$C_7H_6O_3$	138.12	136.95	-	65.0<br>93.0	23	25<br>13	1.88
Protocatechuic acid	$C_7H_6O_4$	154.12	152.95	-	108.95	25	13	1.64
Vanillin	$C_8H_8O_3$	152.15	151.0	-	92.2<br>136.0	22	20<br>15	2.23
p-hydroxy benzaldehyde	$C_7H_6O_2$	122.13	120.95	-	91.85	12	20	4.69
Gentisic acid	$C_7H_6O_4$	154.13	153.05	-	108.2	13	25	4.28
				**Gallic acid derivatives**				
Gallic acid	$C_7H_6O_5$	170.12	168.95	-	78.98<br>124.95	23	22<br>15	1.37
Ethyl gallate	$C_9H_{10}O_5$	198.18	197.05	-	124.0	15	25	4.66
Syringic acid	$C_9H_{10}O_5$	198.17	197.0	-	122.95<br>182.0	27	23<br>13	1.93
				**Cinnamic acid derivatives**				
Ferulic acid	$C_{10}H_{10}O_4$	194.18	192.95	-	134.0<br>178.0	26	25<br>12	2.20
Ferulic acid ethyl ester	$C_{12}H_{14}O_4$	222.24	223.2	+	177.15	15	13	5.84
Caffeic acid	$C_9H_8O_4$	180.16	178.95	-	134.95	25	13	1.89
Dihydro caffeic acid	$C_9H_{10}O_4$	182.17	181.05	-	137.05	22	12	4.39
Trans-cinnamaldehyde	C_9H_8O	132.16	133.15	+	55	20	11	5.93
Trans-cinnamyl alcohol	$C_9H_{10}O$	134.18	117.25	-	115	18	17	5.45
m-coumaric acid	$C_9H_8O_3$	164.16	163.1	-	118.9	20	12	4.81
p-coumaric acid	$C_9H_8O_3$	164.16	163.0	-	119.0	15	13	2.13
Chlorogenic acid	$C_{16}H_{18}O_9$	354.31	353.1	-	84.0<br>191.02	22	44<br>14	1.70
				**Coumarin derivatives**				
Coumarin	$C_9H_6O_2$	146.15	147.01	+	91<br>102.9	17	12<br>17	5.44
m-hydroxycoumarin	$C_9H_6O_3$	162.15	161.15	-	133	17	13	5.26
p-hydroxycoumarin	$C_9H_6O_3$	162.15	163.0	+	121	39	13	5.04
7-hydroxycoumarin	$C_9H_6O_3$	162.15	163.15	+	107.2	20	21	4.97

Osthol	$C_{15}H_{16}O_3$	244.29	245.18	+	131.1<br>189	14<br>14	21<br>13	2.30
**Phenolic derivative**								
Eugenol	$C_{10}H_{12}O_2$	164.21	165.25	+	123.95	14	15	6.08
**Furanocoumarin derivatives**								
Isopimpinellin	$C_{13}H_{10}O_5$	246.22	247.15	+	217.1	27	24	5.93
Xanthotoxin	$C_{12}H_8O_4$	216.19	217.15	+	161.2<br>202.1	15	20<br>25	5.75
Xanthotoxol	$C_{11}H_6O_4$	202.17	203.15	+	131.15<br>147.05	22	18<br>21	5.09
**Flavanones derivatives**								
2'-hydroxyflavanone	$C_{15}H_{12}O_3$	240.27	239	-	119.3<br>93.1	40	25<br>16	3.42
7-hydroxyflavanone	$C_{15}H_{12}O_3$	240.27	239.05	-	135.2<br>91.15	41	25<br>23	3.42
4'-methoxyflavanone	$C_{16}H_{14}O_3$	254.29	255.15	+	240<br>161.3	31	17<br>22	3.78
Naringin	$C_{22}H_{32}O_{14}$	580.54	579.15	-	271.1<br>151.5	45	33<br>40	2.21
**Flavone derivatives**								
Apigenin	$C_{15}H_{10}O_5$	270.05	269.15	-	117	31	29	5.24
Apigenin-7-*O*-glucoside	$C_{21}H_{20}O_{10}$	432.38	431.15	-	268.35	35	22	2.15
Luteolin	$C_{15}H_{10}O_6$	286.25	285.13	-	133.25	24	37	4.99
Luteolin-7-*O*-glucoside	$C_{21}H_{20}O_{11}$	448.38	449.15	+	287.1	34	31	2.01
**Flavonol derivatives**								
Isorhamnetin	$C_{16}H_{12}O_7$	316.28	315	-	151.0<br>300.2	43	30<br>20	2.86
Quercetin-3-*O*-rhamnoside	$C_{21}H_{20}O_{11}$	448.38	447.01	-	271.0<br>300.0	43	47<br>28	2.14
Quercetin-3-*O*-rutinoside	$C_{27}H_{30}O_{16}$	610.53	609.1	-	300.0<br>271	47	39<br>65	1.92
Quercetin-3-O-galactoside	$C_{21}H_{20}O_{12}$	464.38	463.3	-	300.0<br>271.15	47	24<br>44	1.99
Myricetin-3-*O*-galactoside	$C_{21}H_{20}O_{13}$	480.38	479.05	-	271.1<br>287.1	48	39<br>44	1.87
Myricetin-3-*O*-rhamnoside	$C_{21}H_{20}O_{12}$	464.38	463.15	-	316.3<br>271.25	31	27<br>42	4.38
Kaempferol	$C_{15}H_{10}O_6$	286.25	285.25	-	151	30	14	5.32

Kaempferol-3-*O*-rutinoside	$C_{21}H_{20}O_{10}$	432.39	431.05	-	255.3<br>284.2	45	42<br>28	2.27
**Catechins and Procyanidins**								
Procyanidin-B2	$C_{30}H_{26}O_{12}$	578.53	577	-	125.05<br>289.3<br>407.35	29	32<br>23<br>24	2.30

Table S5: The limit of detection (LOoD), quantification (LoQ), linearity, precision, and accuracy results for the screened polyphenolic compounds contained in *E. officinalis.* The calibration equations represent the peak area as a function of concentration in ppb. The intra- and inter-day experimental data concern data that have been collected from a five-day experiment, whereas the %recovery data are the means of three independent experiments.

Compound	Linear range (ppb)	LOD (ppb)	LOQ (ppb)	Calibration equation	Correlation coefficient (r^2)	%RSD		%REC
						(intra-day)	(inter-day)	
**Benzoic acid derivatives**								
m-hydroxybenzoic acid	13.39-499.21	13.39	40.58	y=42.16x+277.25	0.9997	1.00	2.06	99.9
p-hydroxybenzoic acid	3.01-499.50	3.01	14.20	y=36.87x-62.07	0.9991	1.15	2.21	98.8
Protocatechuic acid	0.66-504.50	0.66	14.70	y=34.24x-69.4	0.9995	1.25	2.65	86.3
Vanillin	2.87-335.00	2.87	5.62	y=0.67x-0.1	0.9999	0.98	0.95	100.4
p-hydroxy benzaldehyde	0.57-99.46	0.57	1.72	y=7.43x+36.81	0.9991	3.21	3.25.	95.6
Gentisic acid	7.06	7.06	21.40	y=3.40x+36.81	0.9994	2.47	2.6	100.3
**Gallic acid derivatives**								
Gallic acid	53.20-513.20	53.20	105.20	y=0.67x-1.5	0.9996	0.46	0.21	99.9
Ethyl gallate	1.21-108.36	1.21	3.69	y=21.07x+123.35	0.9999	1.06	1.33	97.5
Syringic acid	2.01-501.60	2.01	2.86	y=7.28x-2.7	0.9996	1.36	1.01	96.6
**Cinnamic acid derivatives**								
Ferulic acid	2.10-505.60	2.10	12. 17	y=19.02x-68.4	0.9992	0.7	2.45	102.6
Ferulic acid ethyl ester	0.11-498.50	0.11	0.32	y=2013.99x+16320.9	0.9994	3.21	3.69	93.6
Caffeic acid	1.21-500	1.21	1.25	y=92.95x+344.4	0.9995	1.01	2.21	100.1
Dihydrocaffeic acid	97.28-499.66	97.28	294.81	y=11.92+64.06	0.9998	0.97	1.14	94.4
Trans-cinnamaldehyde	0.76-500.6	0.76	2.30	y=270.02x+832.24	0.9997	1.59	2.63	93.2
Trans-cinnamyl alcohol	1.37-498.14	1.37	4.16	y=82.34x+552.83	0.9993	2.24	2.59	99.9
p-coumaric acid	0.65-497.30	0.65	1.55	y=52.84x+36.9	0.9997	1.7	1.94	93.2

m-coumaric acid	1.21-497.70	1.21	2.87	Y=70.92x+473.76	0.9996	1.64	2.24	97.8
Chlorogenic acid	3.48-495.60	3.48	4.76	y=25.02x+60.3	0.9991	1.35	1.98	87.4
**Coumarin derivatives**								
Coumarin	0.91-497.55	0.91	2.76	y=2370.35x+15986.5	0.9997	2.36	3.62	92.1
m-hydroxycoumarin	11.84-503.71	11.84	35.90	y=2.59x+22.96	0.9970	1.34	3.01	97.8
p-hydroxycoumarin	1.06-498.01	1.06	3.21	y=1104.02x+9957.32	0.9992	2.21	2.59	96.1
7- hydroxycoumarin	1.98-498.10	1.98	6.02	Y=62.10x+907.80	0.9990	1.94	2.46	99.24
Osthol	0.11-499.80	0.11	0.3	y=1772.94x+30399.4	0.9999	1.36	2.15	95.7
**Phenolic derivative**								
Eugenol	3.63-497.40	3.63	11.01	y=89.12x+643	0.9987	2.25	4.68	101.2
**Furanocoumarin derivatives**								
Isopimpinellin	0.45-96.16	0.45	14.85	y=2987.59x+3078.12	0.9975	3.21	4.12	98.4
Xanthotoxin	0.30-496.65	0.3	0.9	y=983.972x+13022.7	0.9996	2.69	3.62	101.2
Xanthotoxol	0.27-498.16	0.27	0.81	y=1276.71x+9756.89	0.9992	1.58	2.21	95.5
**Flavanone derivatives**								
2’-hydroxyflavanone	19.50-250.00	19.50	20.12	y=38.69x+22.5	0.9998	2.7	4.32	99.5
7-hydroxyflavanone	1.97-249.90	1.97	2.21	y=51.17x-73.6	1	2.63	1.42	98.9
4’-methoxyflavanone	2.21-250.00	2.21	3.89	y=83.54x+60.3	0.9999	2.89	1.87	93.6
Naringin	3.01-250.60	3.01	1.21	y=22.88x-43.3	0.9997	2.22	4.02	95.4
**Flavone derivatives**								
Apigenin	1.53-499.98	1.53	4.66	y=64.17x+735.75	0.9998	1.36	2.47	97.7
Apigenin-7-*O*-glucoside	1.87-125.30	1.87	4.42	y=6.17x+3.8	0.9998	3.48	2.54	95.8
Luteolin	5.85-499.37	5.85	17.73	y=10.02x+123.29	0.9997	1.11	2.21	99.1
Luteolin-7-*O*-glucoside	2.21-250.10	2.21	2.22	y=51.52x-89.9	0.9998	3.64	3.22	89.2
**Flavonol derivatives**								

Isorhamnetin	14.01-251.1	14.01	2.31	y=6.08x-15.4	0.9992	2.48	1.18	100.1
Quercetin-3-*O*-rhamnoside	1.02-250.60	1.02	4.21	y=60.83x-38.6	0.9999	2.21	3.01	99.8
Quercetin-3-*O*-rutinoside	1.40-251.30	1.40	4.32	y=97.74x+109.7	0.9999	1.35	1.89	87.4
Quercetin-3-*O*-galactoside	6.32-249.90	6.32	3.21	y=3.97x+0.5	0.9998	2.14	1.37	96.3
Myricetin-3-*O*-rhamnoside	3.12-500.00	3.12	9.47	y=17.99x+80.65	0.9997	1.10	2.01	100.6
Myricetin-3-*O*-galactoside	0.85-251.20	0.85	2.12	y=26.38x-31.8	0.9997	1.78	1.65	100.2
Kaempferol	10.61-499.28	10.61	32.16	y=2.59543x+14.47	0.9995	2.32	2.69	89.9
Kaempferol-3-*O*-rutinoside	0.76-250.00	0.76	1.21	y=25.73x+73.7	0.9997	1.36	2.21	91.2
**Catechins and Procyanidins**								
Procyanidin-B2	2.04-498.94	2.04	6.18	y=10.76x+42.68	0.9998	2.36	3.12	96.3

SIR ESI-(±)-DICHLOROMETHANE FRACTION

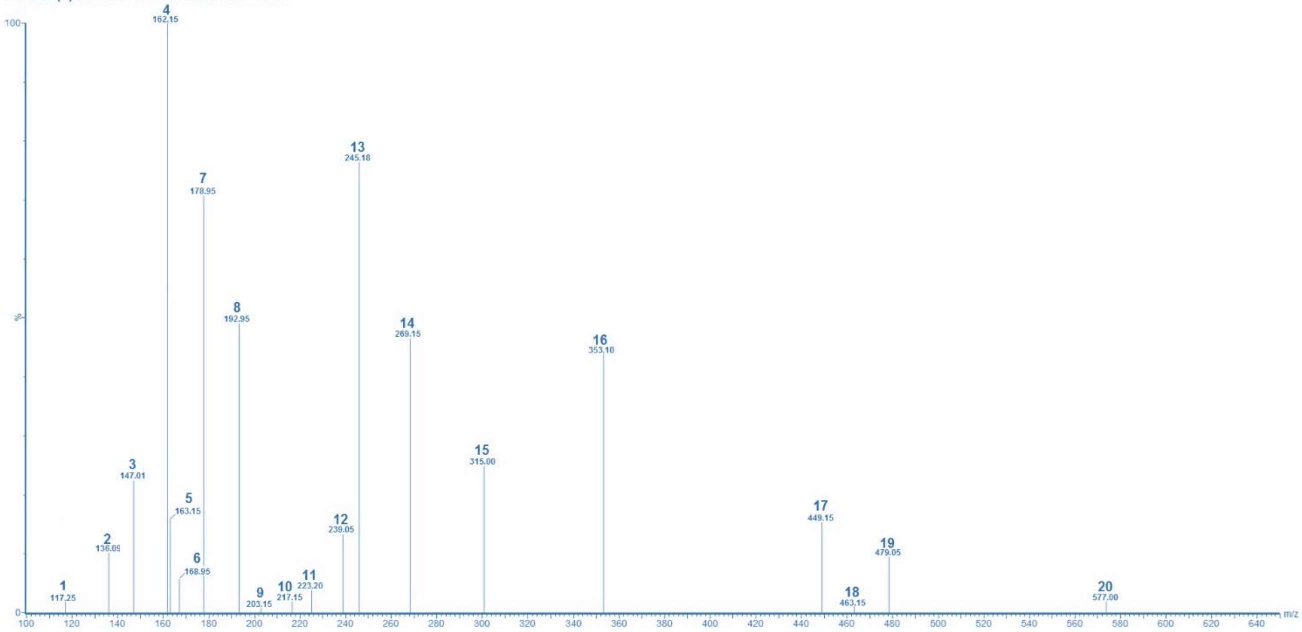

SIR ESI-(±) METHANOL FRACTION

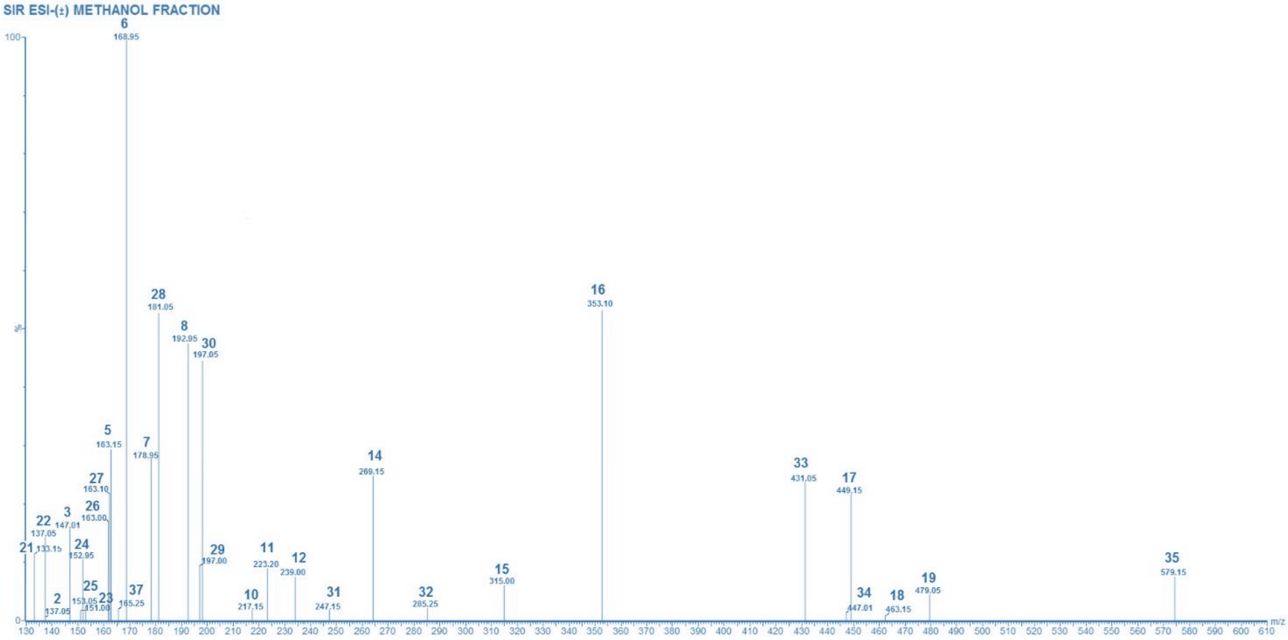

SIR ESI-(±) WATER (1) FRACTION

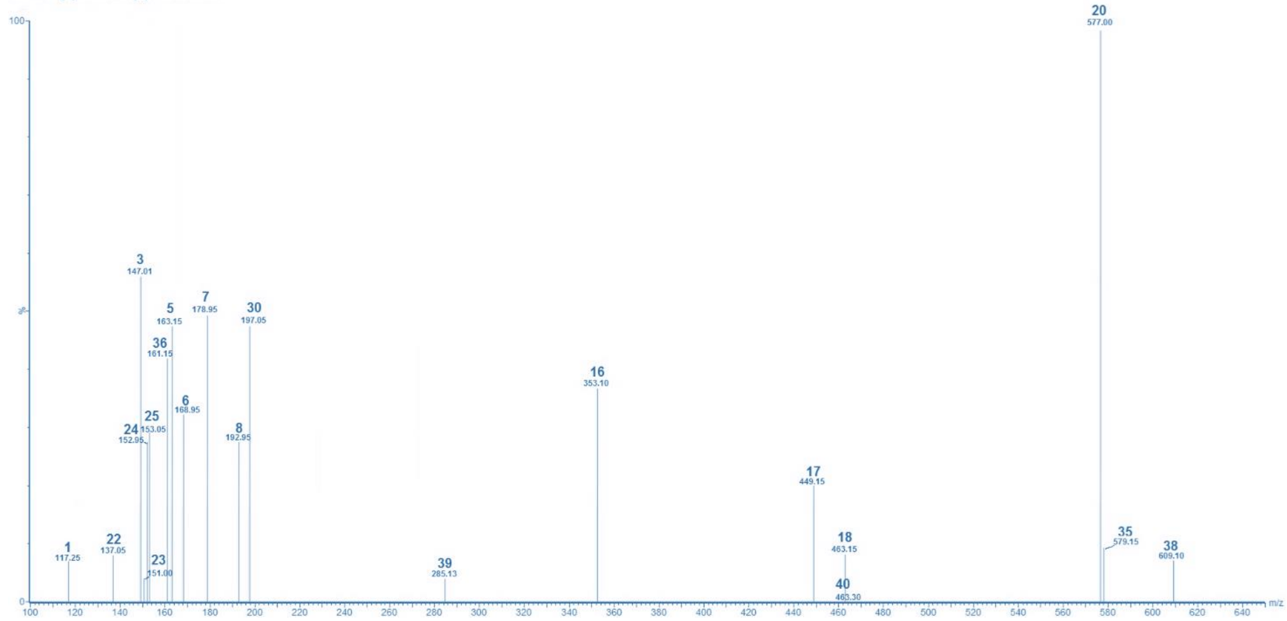

SIR ESI-(±) DIETHYL ETHER FRACTION

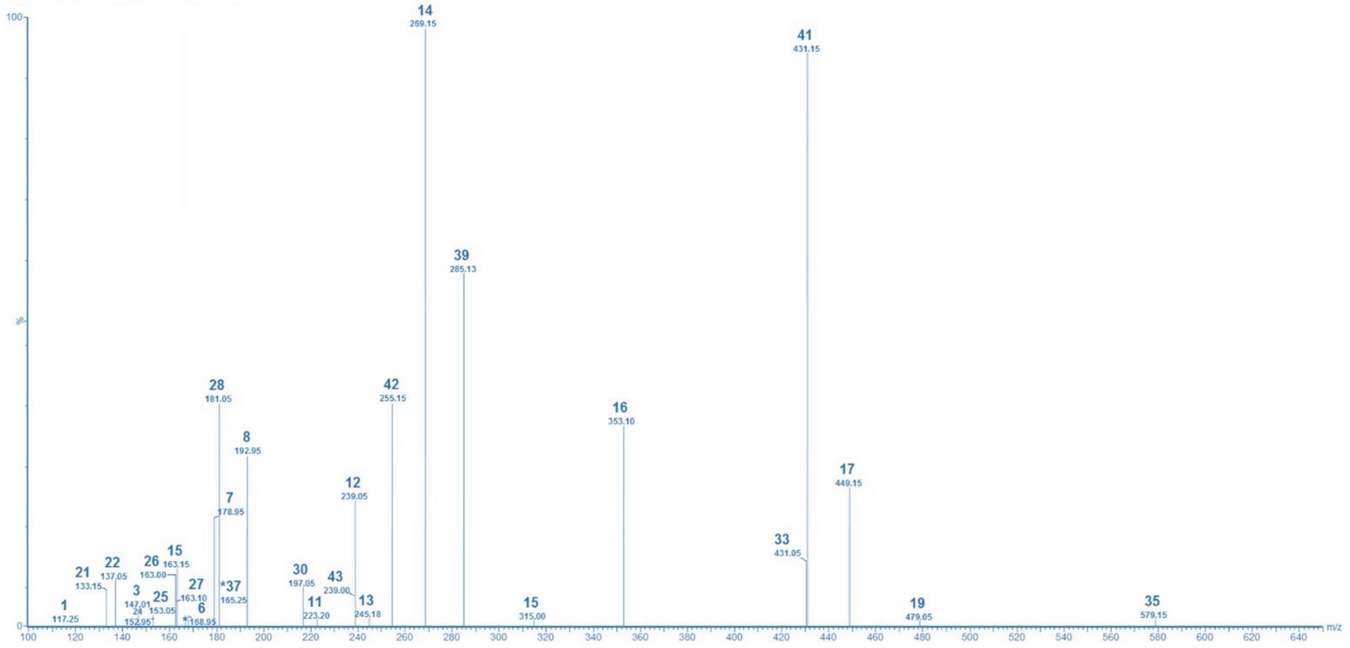

SIR ESI-(±) ETHYL ACETATE FRACTION

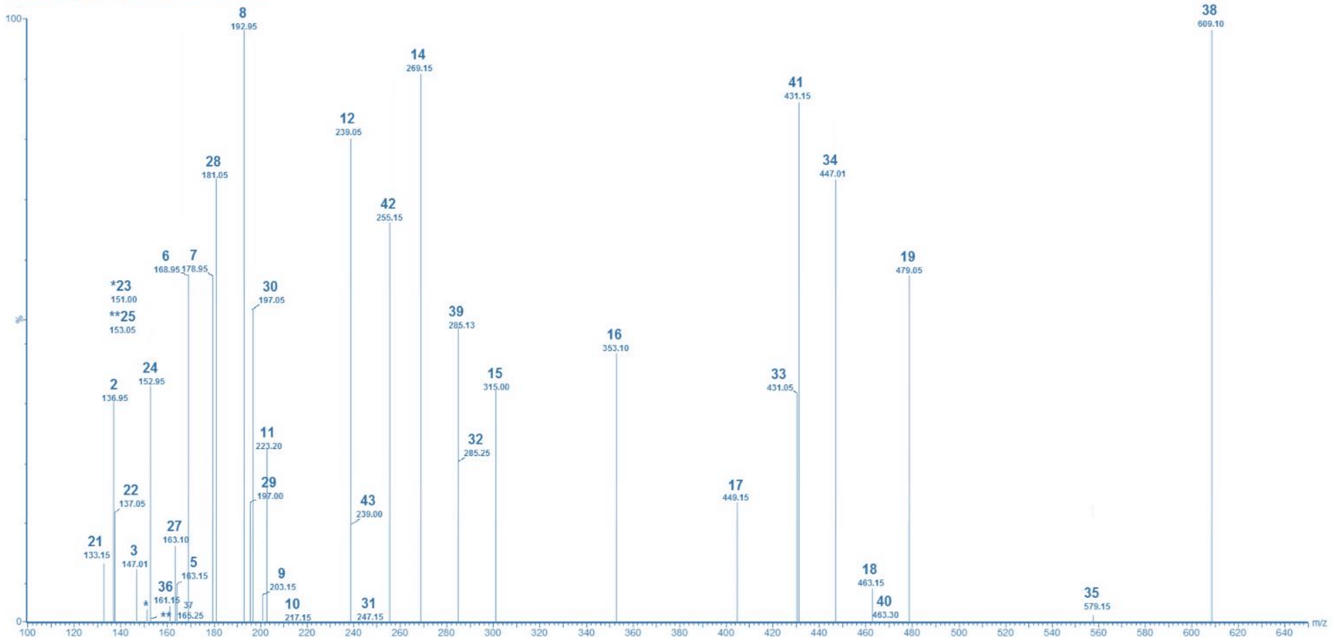

SIR ESI-(±) BUTANOL FRACTION

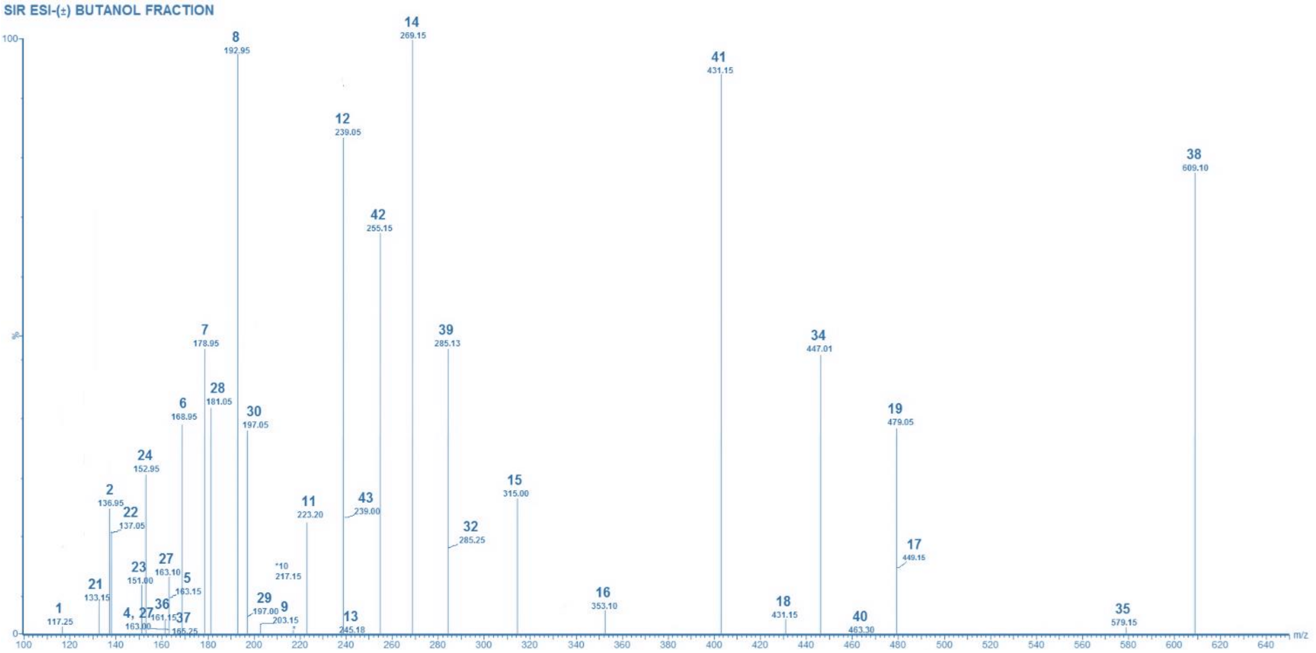

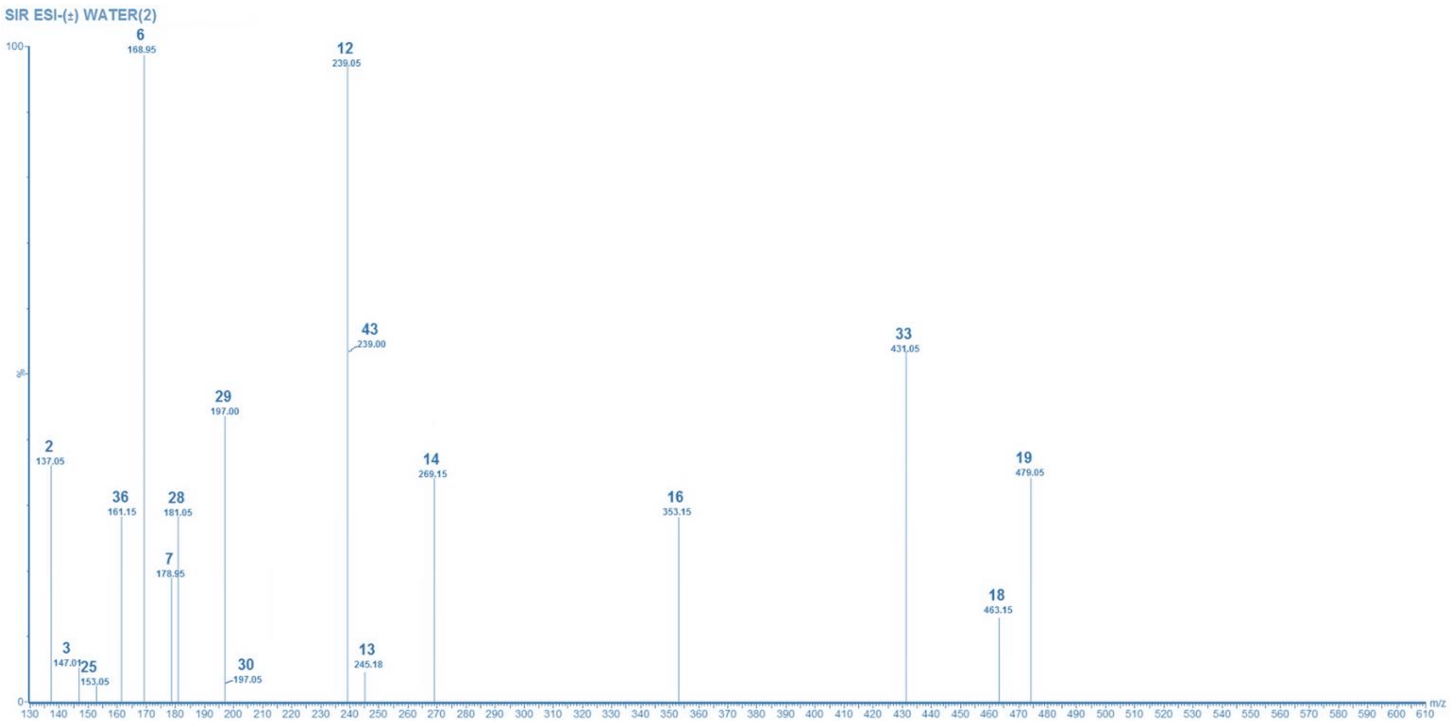

Figure S1: The Selected Ion Recording (SIR) spectrum of all the fractions (dichloromethane, methanol, water-1, diethyl ether, ethyl acetate, butanol and water-2) ionised under both positive and negative electrospray ionisation (±ESI); **1** – trans cinnamyl alcohol, **2** – *p*-hydroxy benzoic acid, **3** – coumarin, **4** – p-hydroxycoumarin, **5** – 7-hydroxycoumarin, **6** – gallic acid, **7** – caffeic acid, **8** – ferulic acid, **9** – xanthotoxol, **10** – xanthotoxin, **11** – ferulic acid ethyl ester, **12** – 7-hydroxyflavanone, **13** – osthol, **14** – apigenin, **15** – isorhamnetin, **16** – chlorogenic acid, **17** – luteolin-7-*O*-glucoside, **18** – myricetin-3-*O*-rhamnoside, **19** – myricetin-3-*O*-galactoside, **20** – procyanidin-B2, **21** – *trans*-cinnamaldehyde, **22** – *m*-hydroxy benzoic acid, **23** – vanillin, **24** – protocatechuic acid, **25** – gentisic acid, **26** – *p*-coumaric acid, **27** – m-coumaric acid, **28** – dihydrocaffeic acid, **29** – syringic acid, **30** – ethyl gallate, **31** – isopimpinellin, **32** – kaempferol, **33** – kaempferol-3-O-rutinoside, **34** – quercetin-3-*O*-rhamnoside, **35** – naringin, **36** – m-hydroxycoumarin, **37** – eugenol, **38** – quercetin-3-*O*-rutinoside, **39** – luteolin, **40** – quercetin-3-*O*-galactoside, **41** – apigenin-7-*O*-glucoside, **42** – 4’-methoxyflavanone, **43** – 2’-hydoxyflavanone.
